# Indurated erythema of abdominal skin: An unusual presentation of metastatic endometrial carcinoma—Case report with literature review

**DOI:** 10.1002/ski2.136

**Published:** 2022-06-05

**Authors:** Dennis Niebel, Paula Kranert, Mark Berneburg, Konstantin Drexler, Maria Isabel von Eichborn, Jan Braess, Michael Allgäuer, Sigrid Karrer

**Affiliations:** ^1^ Department of Dermatology University Hospital Regensburg Regensburg Germany; ^2^ Clinic for Oncology and Hematology Krankenhaus der Barmherzigen Brüder Regensburg Germany; ^3^ Clinic for Radiation Therapy Krankenhaus der Barmherzigen Brüder Regensburg Germany

## Abstract

Carcinoma erysipelatoides (CE) is a rare clinical manifestation of cutaneous metastasis, which mimics inflammatory conditions such as erysipelas. Depending on the site of the originating tumour, unusual manifestations involving different sites of the body may occur. We herein report a case of a 60‐year‐old female patient with metastatic endometrial carcinoma presenting as CE of the abdominal skin and the inguinal folds. Even though the diagnosis of advanced malignancy had been established before and she was currently receiving chemotherapy (carboplatin and paclitaxel), the clinical appearance closely resembled fungal (candidal intertrigo) and consecutively bacterial (erysipelas) infection, which resulted in treatment with antimycotics and antibiotics at first. Dermatohistopathological examination of skin biopsies revealed a diffuse and nodular infiltrate of pleomorphic atypical tumour cells with strong expression of cytokeratin 7 and PAX8, also detectable within lymphatic vessels. Therapy comprised antiseptic ointments to prevent superinfection, palliative electron beam radiation and supportive care. Since there were no targetable KRAS‐, NRAS‐ and BRAF‐gene mutations, systemic therapy was switched to checkpoint inhibition (pembrolizumab) in combination with lenvatinib. The overall prognosis of cutaneous metastasis of endometrial carcinoma is dismal with most patients succumbing to disease within few months. Similarly, our patient died after 3 months due to sepsis in the course of malignant pleural effusion. We aim to highlight the possibility of unusual sites of CE and the risk of respective clinical misdiagnoses.

## Graphical abstract

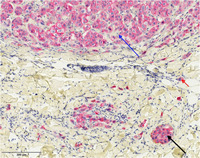

1

**What is already known about this topic?**

- Cutaneous metastatic disease is clinically heterogeneous, it most often appears as singular or multiple nodules.^1^ Carcinoma erysipelatoides (CE) is another manifestation and commonly arises in the thoracic region^2^; numerous solid tumours may be the precursor, breast cancer being the most common one.^3^ Very rarely, CE affects abdominal skin.^4^.
- Endometrial carcinoma shows cutaneous metastasis in only 0.8–1% of the patients.^5^ As a late presentation of metastatic disease, the prognosis is dismal and treatment is tailored on an individual basis.

**What does this study add?**

- This case report adds illustrative clinical and histopathological images of a very rare presentation of endometrial CE. We aim to highlight the potential of misdiagnosis as the infiltration of lymphatic vessels typically renders an inflammatory appearance that may be mistaken for erysipelas or mycotic infections.

## 2 INTRODUCTION

Carcinoma erysipelatoides (CE) commonly affects the thoracic region.^2^ However, depending on the primary tumour and the regional extent of disease, unusual manifestations involving all body regions may occur.^3^ We herein report a case of endometrial carcinoma metastatic to abdominal skin that was treated with antimycotics and antibiotics due to a clinical picture mimicking infection.

## 3 CASE REPORT

**FIGURE 1:**
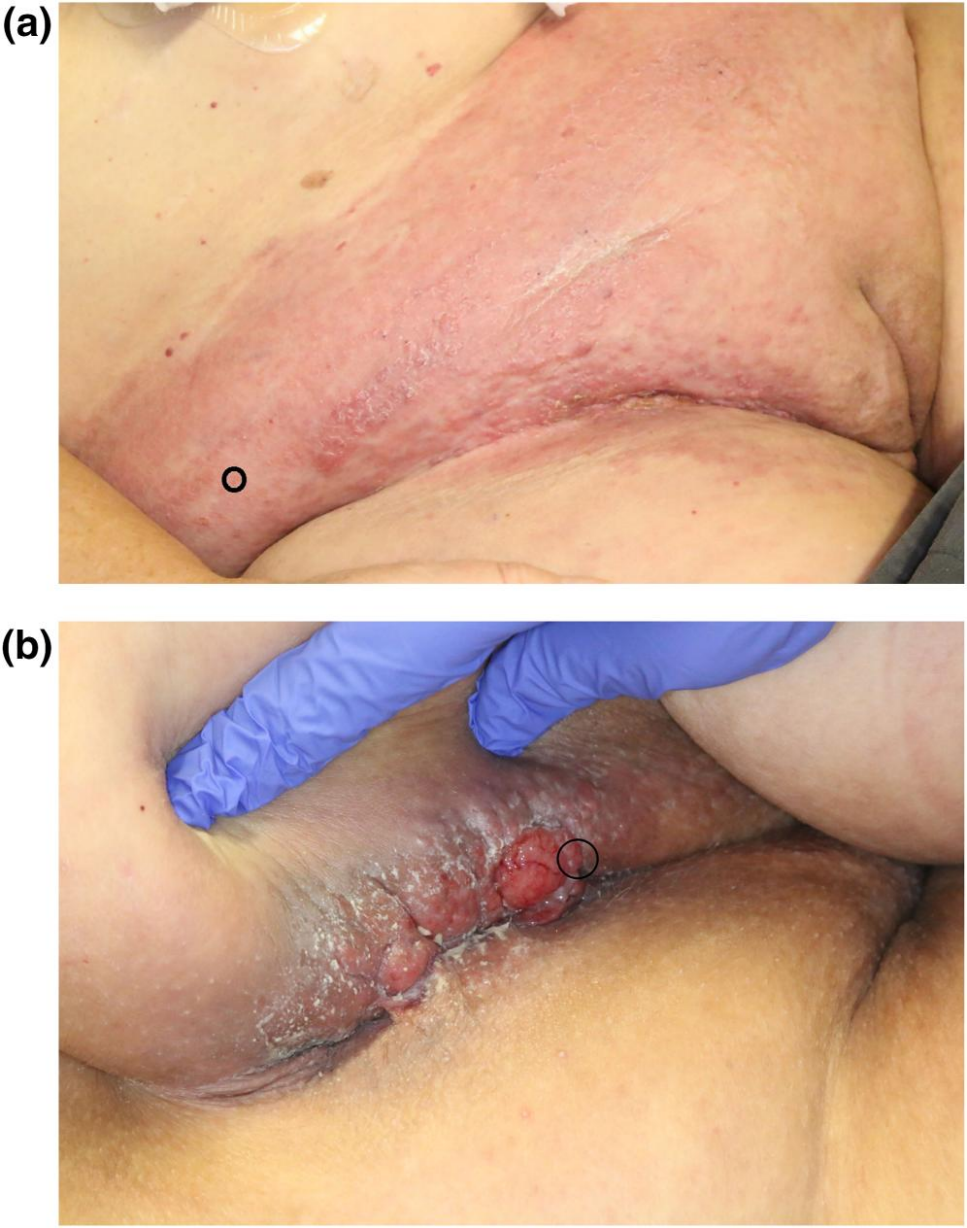
Clinical findings. (a) Erythema and skin thickening of the lower abdominal region with visible small papules. (b) Erythema and skin thickening with papules and one weeping nodule of the left inguinal fold. Note the remnants of bifonazole paste. Biopsy sites are highlighted with a black circle

A 60‐year‐old female patient was referred to our Dermatological department with a diffusely demarcated slightly scaling erythema, thickening of abdominal skin (peau d'orange‐like appearance) and sloughing of the intertriginous areas (Figure 1a). Upon close inspection of the skin folds, several papules and one weeping nodule were apparent (Figure 1b). She had already received nystatin paste and later fluconazole 200 mg per os once daily without improvement. With increasing warmth over the area, the patient had received amoxicillin and clavulanic acid 875 mg/125 mg twice daily for a week without improvement. The topical treatment had been switched to bifonazole paste upon which the erythema had first become papulous.

**FIGURE 2:**
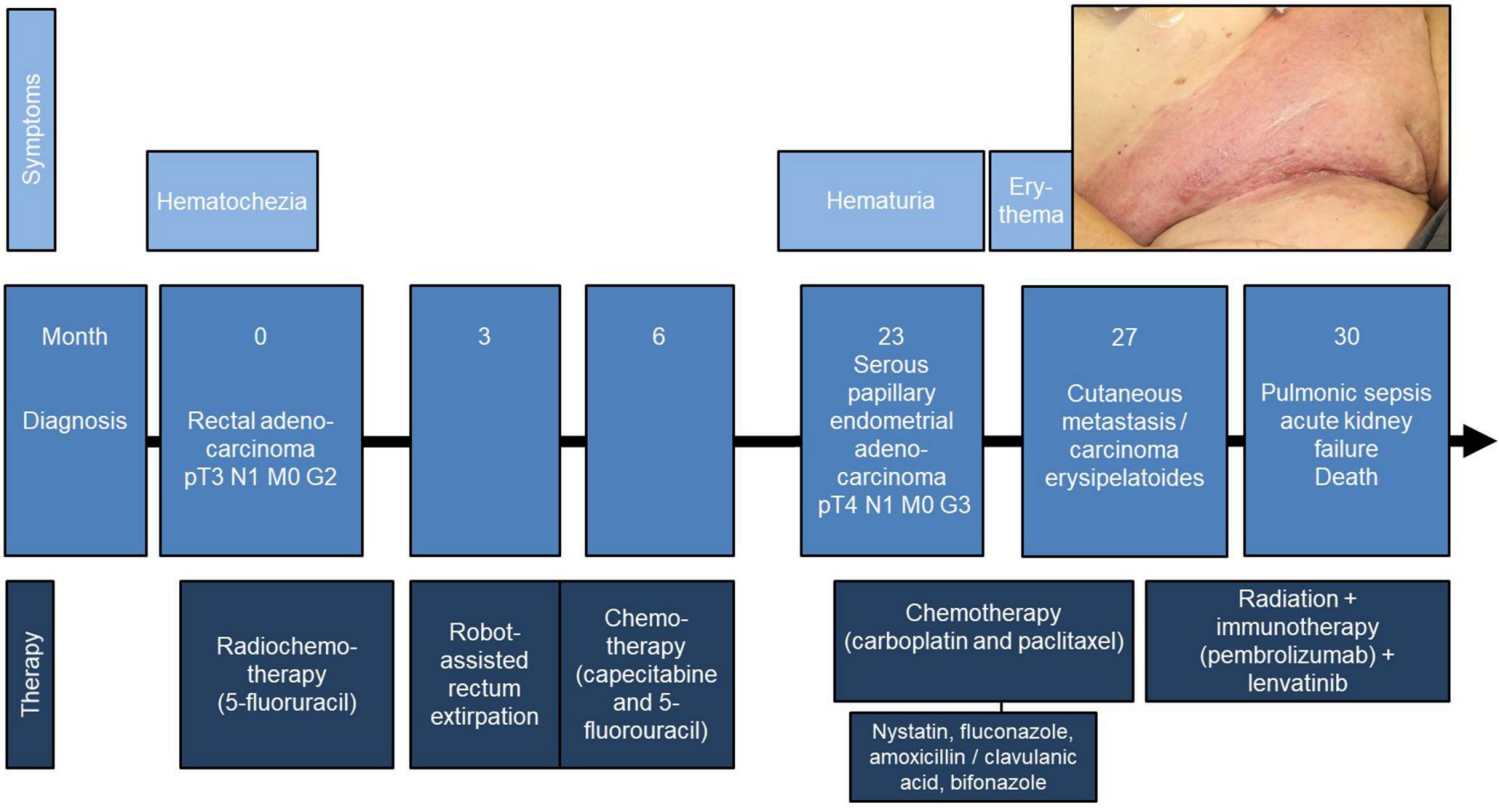
Timeline of the events including symptoms, diagnoses and treatments

Two years earlier, a supra‐anal rectal adenocarcinoma had been managed with robot‐assisted rectum extirpation (pT3 pN0 L0 V0 Pn0 cM0 G2) after 3 months of neoadjuvant radiochemotherapy (5‐fluorouracil) (Figure 2). Upon successful surgery in curative intention, adjuvant chemotherapy (capecitabine and 5‐fluorouracil) had been continued for another 3 months. Quarterly staging examinations had been unremarkable for more than a year. However, 4 months prior to the consultation in our clinic, an advanced serous papillary adenocarcinoma of the endometrium (low‐grade) had been diagnosed after medical examination of a sudden onset of haematuria. The tumour showed extension to cervix uteri, vagina and bladder and locoregional lymph node metastases had been present at time of diagnosis (cT4 cN1). After interdisciplinary assessment, a carboplatin/paclitaxel‐based chemotherapy had been initiated with palliative intention. The patient was continuing this treatment at the point of referral to our clinic.

**FIGURE 3:**
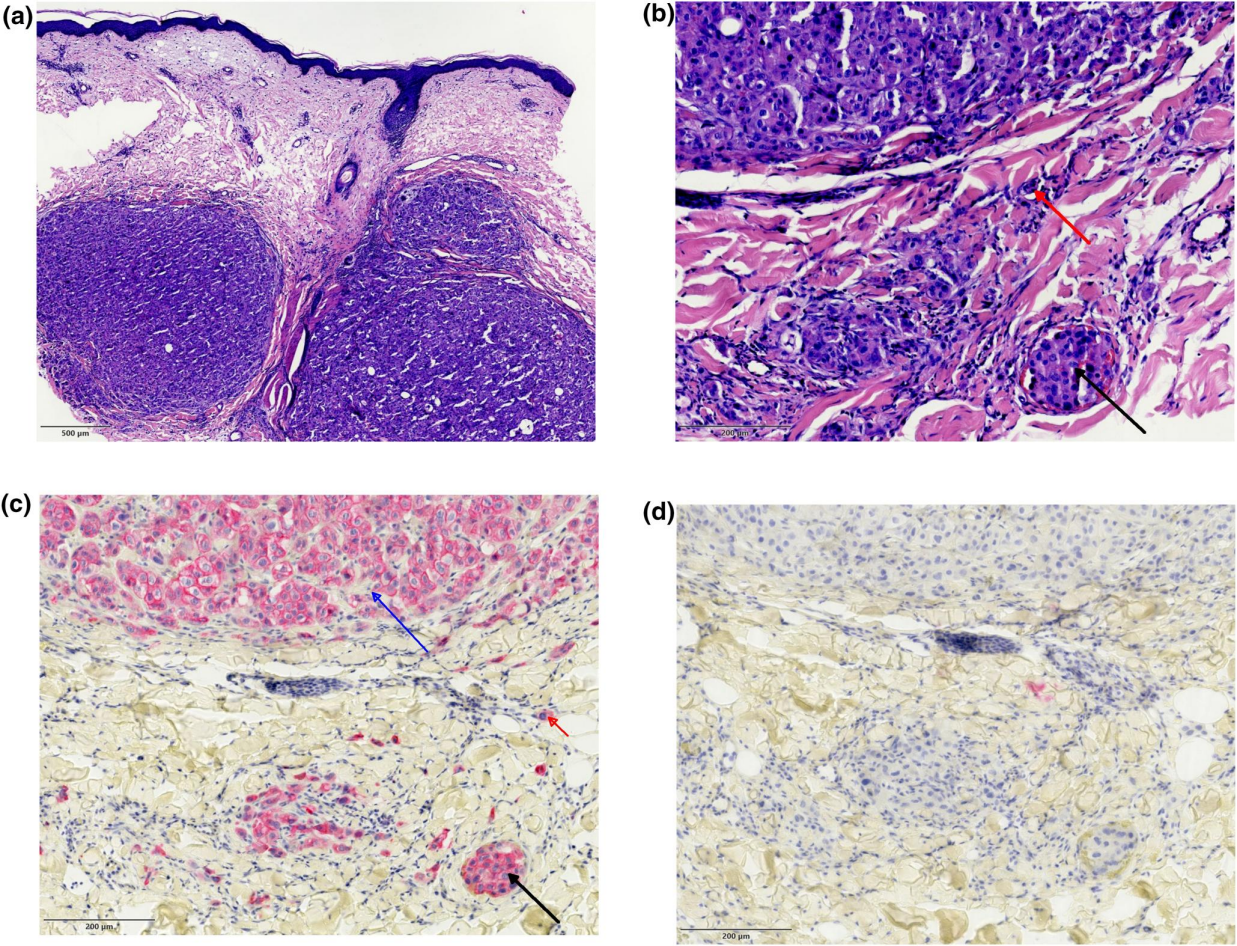
Histopathologic findings. (a) Punch biopsy of weeping nodule of the left inguinal fold corresponding to Figure 1b: nodular and diffuse infiltrates of pleomorphic atypical tumour cells (H & E, 10× original magnification, scale bar indicating 500 μm). (b) Enlargement of an area within the deep dermis. Both intralymphatic (red arrow) and intravascular tumour cells (black arrow) are visible (H & E, 40× original magnification, scale bar indicating 200 μm). (c) Consistent expression of cytokeratin 7 in the nodular infiltrate (blue arrow), the intralymphatic (red arrow) and intravascular (black arrow) cells (CK7, 40× original magnification, scale bar indicating 200 μm). (d) Absence of cytokeratin 20 expression (CK20×, 40× original magnification, scale bar indicating 200 μm)

Upon clinical inspection we suspected further cancerous spreading to the skin from either or even both of the previously diagnosed cancers (Month 0 rectal carcinoma, month 23 endometrial carcinoma). We performed three punch biopsies from the left inguinal fold, the perianal area and erythematous skin on the right abdominal skin. The specimens displayed diffuse infiltration of poorly differentiated pleomorphic atypical tumour cells and the biopsy from the left inguinal fold showed nodular aggregates of these cells within the dermis (Figure 3a,b). The tumour cells were round‐oval with an eosinophilic cytoplasm and had distinct nucleoli. There were noticeable mitotic figures throughout the tumour, however, no mucous vacuoles in PAS stain whose absence pointed against rectal cancer. To better differentiate between the tumours, an immunohistochemistry panel was added. There was no expression of CDX2, chromogranin A, TTF1 and GATA3, neither a DNA mismatch repair deficiency. As the tumour cells consistently expressed cytokeratin 7 and PAX8 in absence of cytokeratin 20, carcinoembryonic antigen (CEA) and pancytokeratine (Figure 3c,d), we diagnosed endometrial carcinoma metastatic to the skin in all three locations. With expression of PAX8 as a marker of Mullerian differentiation, a relapse of rectal adenocarcinoma in the course of laparoscopy was ruled out.^6^ Malignant cells were also detectable within blood vessels and lymphatic vessels (lymphangiosis carcinomatosa).

We advised the use of antiseptic ointments to prevent superinfection of the inguinal folds; due to missing overexpression of oestrogen and progesteron receptors and absence of KRAS‐, NRAS‐ and BRAF‐gene mutations, no targeted therapy was available. Therefore, systemic therapy was switched to checkpoint inhibition (pembrolizumab) in combination with lenvatinib in addition to local treatment with electron beam radiation. The patient initially responded well and the cutaneous lesions regressed within weeks. However, only 3 months after appearance of cutaneous lesions, she was admitted to the hospital with pulmonic sepsis on grounds of malignant pleural effusion. After a week in intensive care, the patient lastly died of acute kidney failure.

## 4 DISCUSSION

Cutaneous metastasis of internal malignancy is rare and comprises only 1% of all cancer patients^7^ or 10% of patients with metastatic disease.^1^ Similar numbers of 0.8%–1.0% are reported for patients with endometrial carcinoma.^5^, ^8^ The most common clinical manifestations are reddish or skin‐coloured papules and nodules.^9^ CE on the other hand arises due to tumour cells spreading diffusely within lymphatic vessels. Impediment of lymphatic drainage via malignant thrombi promotes progressive lymphoedema and skin thickening. The phenomenon is seen most often on the thoracic wall with adenocarcinoma of the breast or lung as primary tumours.^1^, ^2^, ^3^ CE bears a poor prognosis and ‘inflammatory breast cancer’ is a particularly aggressive disease variant.^10^ ‘Cancer en cuirasse’ is a descriptive term of the most dramatic course of CE that results in circumferential enclosing of the thorax with difficulties in movement and ventilation. There are no exact estimates of the incidence of this condition.^11^ CE less frequently affects abdominal skin and patients with progressive gastric, colorectal or other intraperitoneal organ‐derived cancers are at risk.^4^ The periumbilical region is of special interest given its anatomic connection to the abdominal cavity (Sister Mary Joseph's nodule).^12^ 38 cases of umbilical metastasis of endometrial carcinoma were reported in a nationwide Dutch study over a period of 36 years underlining the importance of this anatomic site.^12^ The lower abdominal region and inguinal folds are even more rarely subject to cutaneous metastatic spread, primary tumours include ovarian, endometrial and cervical cancer in females and prostate cancer in males. The lymphatic drainage of internal genital organs is variable but typically does not involve the inguinal region.^13^ However, contiguous tumoral extension into vagina and urethra may result in alignment to the local lymphatic drainage. We suspect this mode of metastasis in our case. Another hypothesis for cutaneous metastasis of intraperitoneal or genital tumours considers seeding of malignant cells during surgical procedures. This is rather unlikely in our case, considering both the aforementioned immunohistochemical profile of the tumour cells and the relative distance to laparotomy scars after rectum exstirpation.

We carried out a literature search of Pubmed, Web of Science and MEDLINE with the search string [endometrial carcinoma] AND [cutaneous metastasis] [ALL FIELDS] to retrieve 44 results. Excluding rare tumour entities, a total of 22 cases was found eligible to be included in the analysis (Table 1). All patients were in the fifth to eigth decade of life with a high number of patients around 60 years of age. As cutaneous metastasis is a late sign of disease, the prognosis was dismal even when organ site involvement was absent at time of diagnosis. Most patients died within 1 year after establishing the diagnosis, the majority of authors described survival of only a few months. These findings were irrespective of the histological subtype and grading of the tumour. In one of the reports, the authors identified a total of 35 published cases of subcutaneous metastases of uterine cancers dating back to the 19th century.^14^ Unfortunately, histological and clinical details (e.g. outcome) of the patients were not provided. Notably, only one more case of endometrial CE was reported by Chang et al,^15^ which clinically showed overwhelming similarities to our case.

**TABLE 1:** Reports of cutaneous metastatic disease of endometrial carcinoma and clinical presentation including carcinoma erysipelatoides (CE) of the lower abdominal skin as identified by our literature review

Age	Histological subtype	Clinical appearance	Body site	Other metastatic sites at time of cutaneous metastasis	Overall survival (mean, range)	Reference
62	Endometrioid adenocarcinoma, grade 2	Ulcerated papules	Around laparotomy site/abdominal skin extending to the vulva and umbilicus	None	5 months	Atallah et al.^16^
62	Not specified	Multiple large friable exophytic tumours	Around laparotomy site/abdominal skin	Diffuse	‘Shortly thereafter’	Bashline et al.^8^
58	Endometrioid adenocarcinoma, grade 2	Multiple haemorrhagic nodules	Initial surgery site	Bilateral pulmonary metastases, vaginal	‘After the second cycle of chemotherapy’	Baydar et al.^17^
62	Papillary serous carcinoma	Erythematous maculopapular rash and nodular lesions: **Erysipelas carcinomatosa**	Inferior abdominal skin and groin	Inguinal and axillary lymph nodes, pulmonary	2 months	Chang et al.^15^
5 casesMean age 61Range (53–69)	Adenoepidermoid carcinoma (3 cases), mesodermal tumour (2 cases)	Subcutaneous nodules/firm masses up to 4 cm (all cases)	Calf (1 case), head (2 cases), abdominal skin (1 case)Disseminated/axilla, trunk, abdominal skin, inguinal folds (1 case)	Variable: Pulmonary (4 cases) and hepatic (1 case)	Variable: Two patients died within 4 months, three patients alive at the time of the report	Damewood et al.^5^
54	Endometrioid adenocarcinoma, grade 2	Erythematous papular rash	Symmetrical distribution over the trunk and lower abdominal skin	Diffuse lymphadenopathy, bone	7 months	Fan et al.^18^
54	Papillary serous carcinoma, grade 3	Pruritic nodules, partly crusted and ulcerated	Pubic skin, vulva	Bone, hepatic and peritoneal	5 months	Kim et al.^19^
56	Endometrioid adenocarcinoma, grade 1	Singular cystic/nodular papule	Scalp	Bone	3 months	Kushner et al.^20^
67	Clear cell adenocarcinoma, grade 2	Erythema and itching followed by subcutaneous mass	Abdominal skin	Vaginal and lymphatic	Alive at the time of the report	La Fianza et al.^14^
72	Endometrioid adenocarcinoma, grade 1	Multiple subcutaneous nodules	Scalp, extremities and trunk	Diffuse (lymphatic, abdominal organs, retroperitoneal, right pleural effusion)	2 weeks	El M'rabet et al.^21^
60	Endometrioid adenocarcinoma, grade 2	Firm non‐tender nodule	Lower leg	Vaginal nodule, no systemic metastasis	11 months	Ma et al.^22^
45	Adenocarcinoma with adenoacanthoma pattern, grade 2	Well‐circumscribed swelling	Frontal region	Local and distant bone metastasis	6 months	Mustafa et al.^23^
60	Serous papillary adenocarcinoma, grade 3	Erythema and skin thickening, several papules and one weeping nodule: **Erysipelas carcinomatosa**	Abdominal skin and inguinal folds	Inguinal and iliacal lymphatic, malignant ascites and pleural effusion, suspicious renal and lineal lesions	3 months	Niebel et al (present case)
60	Endometrial carcinoma not further specified	Multiple erythematous, infiltrated, non‐tender, firm‐to‐hard papules and nodules	Unilaterally at left flank in zosteriform distribution	Rectum, lymphatic, mesenterial, pulmonary, vertebral bone	2 months	Raghupathy et al.^24^
72	Endometrial serous carcinoma, grade 3	Cutaneous tumoral masses	Inferior abdominal skin and hypogastrium	Bilateral inguinal lymph nodes	Alive at time of the report	Rebega et al.^25^
2 cases Mean age 61Range (50–72)	Endometrial adenocarcinoma, not further specified (both cases)	Singular nodule (both cases)	Periumbilical	Pelvic mass, lymphatic (1 case), none (1 case)	Not reported	Samaila et al.^26^
73	Endometrioid adenocarcinoma, grade 2	Soft, warm, pulsatile mass	Lower leg	None	Not reported	Stonard et al.^27^

*Note*: Unusual uterine tumours such as carcinosarcoma and epitheloid trophoblastic tumour were excluded.

When opting for specific treatment modalities, potential side effects such as risk of ulceration and superinfection should be balanced carefully to avoid worsening of quality of life. A broad variety of cytostatic drugs and different radiation therapy regimens are described in the literature as there are no generally accepted treatment recommendations. Even though our patient was assigned a state‐of‐the‐art immuno‐oncologic treatment approach with additional radiotherapy, the outcome was still fatal months after diagnosis.

In summary, we present a striking case of CE of the abdominal skin and inguinal folds. We aim to highlight the possibility of misdiagnoses (intertriginous candidiasis, erysipelas, eczema, erythrasma) even in the light of established advanced oncologic disease.

## AUTHOR CONTRIBUTIONS

**Dennis Niebel:** Conceptualisation (equal); Data curation (equal); Investigation (lead); Visualisation (lead); Writing – original draft (lead); Writing – review & editing (lead). **Paula Kranert:** Writing – original draft (lead); Writing – review & editing (equal). **Mark Berneburg:** Writing – original draft (equal); Writing – review & editing (equal). **Konstantin Drexler:** Writing – original draft (equal); Writing – review & editing (equal). **Maria Isabel von Eichborn:** Writing – original draft (equal); Writing – review & editing (equal). **Jan Braess:** Writing – original draft (equal); Writing – review & editing (equal). **Michael Allgäuer:** Writing – original draft (equal); Writing – review & editing (equal). **Sigrid Karrer:** Visualisation (equal); Writing – original draft (equal); Writing – review & editing (equal).

## CONFLICT OF INTEREST

Dennis Niebel has been an advisor and/or received speakers’ honoraria or travel expense reimbursements and/or received grants and/or participated in clinical trials of the following companies: Abbvie, Almirall, BMS, Kiowa Kyrin, Novartis, Pfizer, GSK, and MSD. Maria Isabel von Eichborn has been an advisor and/or received speakers’ honoraria or travel expense reimbursements and/or received grants and/or participated in clinical trials of the following companies: Abbvie, Boehringer Ingelheim, Janssen. The remaining authors declare no competing financial interest.

## ETHICS STATEMENT

The patient in this manuscript has given written informed consent to publication of her case details.

## Data Availability

Data sharing not applicable to this article as no datasets were generated or analysed during the current study.
